# A Delay-Programmed Kinetic-Chain Model for Racket Strokes with a Two-Parameter Mechanical Stretch-Shortening-Cycle Extension

**DOI:** 10.3390/bioengineering13101232

**Published:** 2026-10-21

**Authors:** Sun-Hyun Youn

**Affiliations:** Department of Physics, Chonnam National University, Yongbong-ro 77, Buk-gu, Gwangju 61186, Republic of Korea; sunyoun@jnu.ac.kr

**Keywords:** tennis biomechanics, kinetic chain, release delay, stretch–shortening cycle, two-parameter model, effective stiffness, athlete-specific optimization

## Abstract

Efficient racket acceleration in tennis is commonly attributed to the kinetic chain, but a mechanical model must specify how energy stored at successive joints is released and transferred to the racket. We formulate a minimal planar body–arm–racket model in which each joint stores elastic energy in a latched posture and releases it after an explicit delay. In this framework, optimized delay produces a proximal-to-distal sequence and a superadditive increase in racket-tip speed from 16.6 m s^−1^ for one active joint to 30.2 m s^−1^ for two joints and 43.1 m s^−1^ for three joints, thereby providing a direct mechanical realization of the kinetic chain. The remaining question is how the stretch–shortening cycle (SSC), normally described through complex muscle–tendon physiology, can be incorporated into the same model. We address this by representing SSC action as a joint-level stiffness-modulation block governed by two effective active parameters: a rebound stiffness gain α and a velocity-dependent firing gain β. These parameters are athlete-dependent mechanical descriptors, not universal biological constants. For one illustrative parameter set, SSC engagement raises the peak speed to 46.7 m s^−1^ (approximately 8%), but this percentage changes with α and β. Because the size of an individual’s active SSC contribution is known to vary substantially from athlete to athlete, this percentage is reported only as an illustration and is not itself the result being claimed. The main contribution is a unified mechanical framework in which time-delayed kinetic-chain release and SSC-like enhancement can be optimized together for athlete-specific posture and stroke design, by adding the two parameters α and β on top of the already delay-optimized passive model without modifying its optimization architecture.

## 1. Introduction

High racket-head speed at ball contact is one of the principal mechanical determinants of stroke performance in tennis. In a powerful serve or forehand drive, the racket is not accelerated by wrist action alone: motion originates in the large proximal segments, propagates through the trunk and shoulder, and reaches the racket only after a delayed distal release. This proximal-to-distal organization, commonly termed the kinetic chain, has been documented across a wide range of striking and throwing skills [1,2,3,4,5,6,7,8,9,10,11,12]. From a mechanical point of view, the kinetic chain may be understood as a timed transfer problem: elastic or muscular energy stored in proximal and distal joints must be released in the correct order so that each distal segment fires into a moving base and transfers energy efficiently to the implement. Although it concerns landing rather than racket strokes, recent work also shows that deliberately adjusted joint kinematics can alter joint-energy dissipation and lower-limb loading, underscoring the broader importance of coordinated joint mechanics in sport movement [13]. In racket strokes, therefore, the issue is not only how much energy is stored in each joint, but when that energy is released. If a distal joint fires too early, the racket accelerates before the proximal segments have acquired sufficient angular momentum; if it fires too late, the hitting configuration has already passed. A useful mechanical model of racket motion should therefore make the release delay itself an explicit parameter. Such a model can turn the qualitative idea of the kinetic chain into a quantitative optimization problem: find the firing times that cause the trunk, shoulder, and wrist to pass through their effective equilibrium postures with maximal angular velocity at nearly the same impact frame. The present paper first develops this delay-programmed description of the kinetic chain. Building on our earlier double- and triple-pendulum studies of energy transfer along the arm–racket chain [14,15,16,17], in which the joint torque was a passive restoring torque $\tau_{i}=-k_{i}\theta_{i}$ and release timing emerged implicitly from the coupled dynamics, we treat delay as an explicit control parameter. Each joint can hold its stored elastic energy in a latched state and release it at a freely chosen firing time. For one active degree of freedom, the model reduces to a closed-form energy conversion problem. For two and three active degrees of freedom, direct numerical scans over the delays identify the firing schedule that maximizes racket-tip speed. In this sense, the optimized time-delayed release is not an auxiliary numerical device; it is the mechanical realization of the kinetic chain within the model.

A second issue arises when the stretch–shortening cycle (SSC) is introduced. The SSC is a well-established mechanism by which an active muscle–tendon unit that is stretched immediately before shortening can produce greater work or power than one that shortens alone [18,19,20,21,22,23,24,25]. However, the biological SSC involves tendon recoil, pre-activation, reflex action, and history-dependent force enhancement. Full musculoskeletal simulations can represent such physiology in detail [26,27,28], but they require many muscle-specific parameters and are not easily combined with the simple delay-optimization framework just described. The key question of this paper is therefore not merely whether SSC can increase racket speed, but how SSC effects can be brought into the same mechanical framework that already describes the time-delayed kinetic chain.

We address this question by reducing the mechanically relevant SSC output to a joint-level stiffness-modulation law. The shoulder and wrist may pass through an eccentric pre-stretch and active rebound before firing, but the active part of the response is represented by only two effective parameters: a rebound stiffness gain α and a velocity-dependent firing gain β. These parameters do not claim that the biological SSC literally consists of two variables. Rather, they serve as mechanical coordinates for an athlete’s effective SSC capacity. Once α and β are specified or identified for a player, the same rigid-body model that optimizes release timing can also be used to optimize preparatory posture, racket lag, and the degree to which SSC-like stiffness enhancement contributes to the stroke.

Thus, the structure of the paper is deliberately hierarchical. First, the kinetic chain is constructed mechanically from stored elastic energy and time-delayed joint release. Second, SSC is introduced not as a separate biological model, but as a two-parameter mechanical extension of the same joint-torque algorithm. Section 2.1 formulates the planar body–arm–racket model. Section 3.1 analyzes the delay-programmed kinetic chain without SSC, progressing from one to three active degrees of freedom. Section 2.2 introduces the two-parameter SSC block and its energy bookkeeping. Section 3 shows how SSC-like enhancement can be added to the delay-optimized kinetic chain. Section 4 and Section 5 discuss the engineering implications and conclude.

## 2. Materials and Methods

### 2.1. Planar Three-Segment Swing Model

#### 2.1.1. Coordinates and Segment Parameters

**Figure 1:**
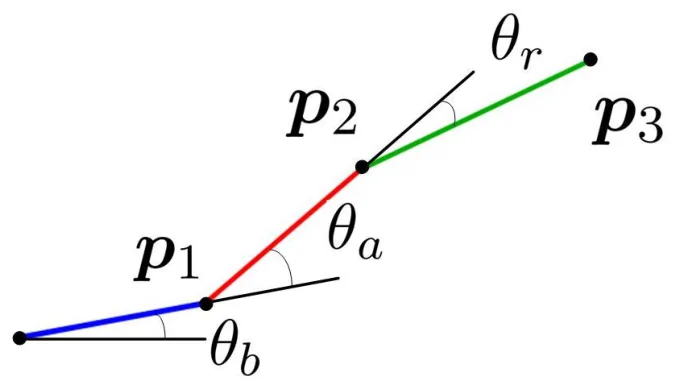
Planar three-segment body–arm–racket model. The representation is deliberately minimal, but it retains the physical ingredients required to study racket-tip speed: segment inertia, geometric leverage, dynamic (inertial) coupling between segments, and the possibility of a delayed distal release.

The swing is modeled as a planar serial chain composed of three rigid segments: body/trunk, arm, and racket (Figure 1). The generalized coordinates are

(1) $$q={\left[\begin{array}{ccc} \theta_{b} & \theta_{a} & \theta_{r} \end{array}\right]}^{T},$$

where $\theta_{b}$ is the absolute body angle, $\theta_{a}$ the arm angle relative to the body, and $\theta_{r}$ the racket angle relative to the arm, so that the absolute arm and racket angles are

(2) $$\phi_{a}=\theta_{b}+\theta_{a},\;\phi_{r}=\theta_{b}+\theta_{a}+\theta_{r}.$$

The segment parameters used in the simulations are listed in Table 1. Each segment is treated as a uniform slender rod with moment of inertia $I_{i}=m_{i}l_{i}^{2}/12$ about its own center of mass. The chosen masses and lengths are broadly consistent with reported adult body-segment anthropometry [29], collapsed into a reduced three-segment trunk–arm–racket representation. This reduction sacrifices anatomical detail (e.g., separate upper-arm and forearm segments) in exchange for a model whose dynamics remain analytically transparent, while preserving the inertial contrast between a heavy proximal segment and a light, long distal implement that is central to the physics of interest.

With the base pivot fixed at the origin, the shoulder, wrist, and racket-tip positions are

(3) $$\begin{array}{cc} p_{1} & =l_{b}\left[\begin{array}{c} \cos\theta_{b}\\ \sin\theta_{b} \end{array}\right],\;p_{2}=p_{1}+l_{a}\left[\begin{array}{c} \cos\phi_{a}\\ \sin\phi_{a} \end{array}\right],\;p_{3}=p_{2}+l_{r}\left[\begin{array}{c} \cos\phi_{r}\\ \sin\phi_{r} \end{array}\right], \end{array}$$

where $p_{3}$, the racket tip, is taken as the impact point.

#### 2.1.2. Racket-Tip Velocity

Differentiating $p_{3}$ gives the racket-tip velocity components

(4) $$\begin{array}{cc} v_{x} & =-l_{b}{\dot{\theta }}_{b}\sin\theta_{b}-l_{a}\left({\dot{\theta }}_{b}+{\dot{\theta }}_{a}\right)\sin\phi_{a}-l_{r}\left({\dot{\theta }}_{b}+{\dot{\theta }}_{a}+{\dot{\theta }}_{r}\right)\sin\phi_{r}, \end{array}$$

(5) vy=−lbθ˙bcosθb+la(θ˙b+θ˙a)cosϕa+lr(θ˙b+θ˙a+θ˙r)cosϕr,

and the scalar racket-tip speed used throughout as the performance measure is $v_{\mathrm{tip}}\;=\;\sqrt{v_{x}^{2}+v_{y}^{2}}$. In the present study, $v_{\mathrm{tip}}$ is evaluated at the modeled impact configuration immediately before ball contact and is used solely as a pre-impact performance indicator. The ball is not included in the equations of motion; consequently, the model does not predict outgoing-ball velocity, spin, or energy transfer during the ball–racket collision.

#### 2.1.3. Equations of Motion

Gravity is neglected because the acceleration phase of interest is short (of order 0.2 s) and the torques generated by the latched springs and by the active joint model of Section 2.2 dominate the gravitational torque by more than an order of magnitude for the parameters used here. The equations of motion take the standard Lagrangian form for a three-link planar chain,

(6) $$M\left(q\right)\ddot{q}+c\left(q,\dot{q}\right)=\tau ,$$

where M(q) is the configuration-dependent mass matrix and $c(q,\dot{q})$ collects the Coriolis and centrifugal terms; the explicit entries are given in Appendix A. The single point of contact between the reduced biomechanics and the rigid-body dynamics is the joint torque vector τ, supplied either by the delay-programmed latched springs of Section 3.1 or by the SSC-augmented torques of Section 2.2. It is this clean interface that makes both mechanisms directly transferable to any general-purpose simulation environment. Equation (6) is integrated numerically (fourth-order Runge–Kutta, fixed step) for all results reported below; the only place an analytic solution is used, as a consistency check, is the single-active-joint case of Section 3.1.2.

#### 2.1.4. Numerical Implementation and Reproducibility

We report here, in full, the integration and search parameters used to produce every numerical result in this paper, exactly as implemented in the simulation code and without alteration for this revision. Equation (6) is integrated with a fixed-step fourth-order Runge–Kutta scheme, step size $h=5\times 10^{-5}$ s, over t∈[0,0.45] s (9000 steps per run); this step is roughly two orders of magnitude smaller than the shortest characteristic joint period implied by the stiffness values of Equation (14). As a consistency check in lieu of a separate step-halving study, the closed-form single-active-joint estimate of Section 3.1.2 agrees with the corresponding numerically integrated peak speed to three decimal places for all three joints (Table 2), which we regard as strong de facto evidence of numerical convergence at this step size. Impact is registered when the active joint angle(s) return to within a fixed posture tolerance of $\theta^{\mathrm{eq}}$: $3^{\circ }$ for one active joint, $5^{\circ }$ for two active joints, and $10^{\circ }$ for three active joints. The optimal release delay of Section 3.1.3 is located by an exhaustive one-dimensional scan of Δt over the entire admissible range [0,0.40] s in 60 steps (≈6.7 ms resolution); the optimal pair reported in Section 3.1.4 is located by an exhaustive two-dimensional 35×35 grid search over $t_{\mathrm{shoulder}}\in \left[0,0.40\right]$ s and $t_{\mathrm{wrist}}\in \left[t_{\mathrm{shoulder}},0.55\right]$ s. Because each scan covers the full physically admissible range at the stated resolution, rather than refining locally around a single initial guess, it functions as a de facto global search; no secondary local maxima in $v_{\mathrm{tip}}$ were found at this resolution in either case. The SSC-enhanced posture search of Section 3 follows the same two-stage logic: a coarse $10^{\circ }$-step grid is scanned over the full admissible initial-posture range, and the five best coarse points are each refined with a $2^{\circ }$-step local grid within $\pm 12^{\circ }$. We have not carried out a systematic (α,β) sensitivity map beyond the single illustrative parameter set of Table 3; extending the search to include α, β, and the pre-stretch velocities as joint optimization variables, together with a full sensitivity map, is a straightforward extension of the present procedure that we leave for future work (Section 4).

### 2.2. Two-Parameter SSC Extension Within the Kinetic-Chain Framework

#### 2.2.1. Physiological Motivation and Modeling Principle

The SSC is a well-established mechanism by which an active muscle–tendon unit that is stretched immediately before shortening can produce greater work or power than one that shortens alone [18,19,20,22,23,24,25]. This enhancement is not attributable to a single cause but to several mechanisms acting in concert: elastic energy storage and recoil in tendon and aponeurosis; a higher force at the onset of shortening due to pre-activation; force enhancement associated with the eccentric loading history; and a neural reflex contribution when the imposed stretch is rapid. That these mechanisms act jointly, at a scale relevant to whole-limb performance, is demonstrated by the classical finding that a countermovement jump outperforms a concentric-only squat jump [30].

The aim here is not to replace the delay-programmed kinetic-chain model with a physiologically detailed SSC model. Rather, the aim is to express the net mechanical contribution of SSC in a form that can be inserted into the same time-delayed joint-torque framework. We therefore adopt the reduced description

(7) $$\tau_{i}≃k_{i,\mathrm{eff}}\left(t,\theta_{i},{\dot{\theta }}_{i},\mathrm{history}\right)\left(\theta_{i}^{\mathrm{eq}}-\theta_{i}\right),$$

in which $k_{i,\mathrm{eff}}$ is not a passive material constant and not an anatomically resolved muscle model. It is an effective, history-dependent mechanical stiffness summarizing the net joint-torque contribution of tendon recoil, pre-activation, reflex action, and related history effects. In this formulation, the active SSC-like part of the response is mapped onto two mechanical parameters: the rebound stiffness gain α and the velocity-dependent firing gain β. Because Equation (7) has the same interface as the passive springs of Section 3.1—a torque as a function of joint state—the SSC block can be added to the kinetic-chain model without changing the equations of motion or the delay-optimization procedure.

#### 2.2.2. Torque Phases of the Active Joint Model

**Figure 2:**
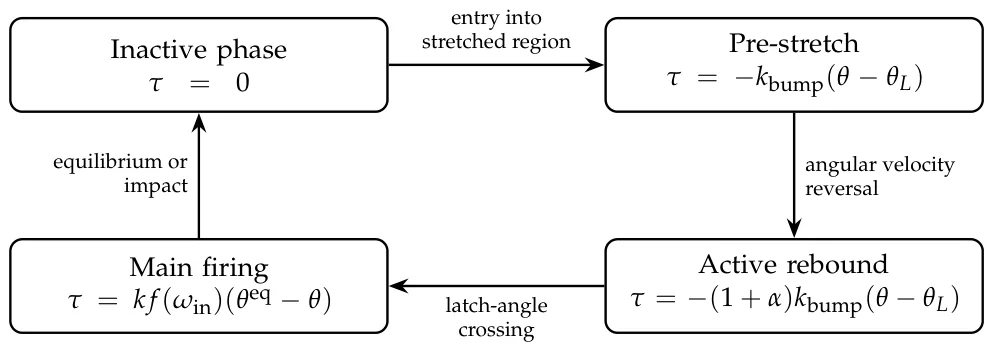
Event-based effective-torque sequence used in the reduced SSC model. The diagram does not represent four independent physical springs; it indicates how the effective joint torque changes as the joint enters the pre-stretch region, reverses its angular velocity, and proceeds to the main firing phase. Because each phase is a torque law of the joint state alone, the entire sequence can be inserted into the same rigid-body simulator used for the delay-programmed kinetic-chain model.

We describe the shoulder and wrist torque $\tau (\theta ,\dot{\theta })$ as passing through four physically motivated phases: *free*, *pre-stretch*, *active rebound*, and *main firing* (Figure 2). Each phase is defined by an explicit torque expression, so that the point at which mechanical energy is injected beyond what a purely passive spring would supply is stated unambiguously.

Before the joint angle reaches the latch region, no elastic torque acts,

(8) $$\tau_{\mathrm{free}}=0,$$

corresponding to the unloaded part of the preparatory motion, prior to engagement of the muscle–tendon unit against its series-elastic limit.

Once θ moves past the latch angle $\theta_{L}$ into the stretched region, a stiff bump spring resists further stretch,

(9) $$\tau_{\mathrm{stretch}}\left(\theta \right)=-k_{\mathrm{bump}}\left(\theta -\theta_{L}\right),$$

representing the combined passive loading of tendon and connective tissue and an initial level of active muscle tension during the eccentric phase, i.e., while the joint is being decelerated at the end of the backswing.

The transition to the genuinely active part of the model occurs at the instant the angular velocity reverses sign—the moment the joint begins to shorten. At that instant the effective stiffness of the bump spring is increased by a factor (1+α),

(10) $$\tau_{\mathrm{rebound}}\left(\theta \right)=-\left(1+\alpha \right)\;k_{\mathrm{bump}}\left(\theta -\theta_{L}\right),$$

which represents the active rebound component of the reduced model. The parameter α is therefore a mechanical gain, not a claim about a single physiological source of the SSC. It summarizes, at the joint level, the combined effect of pre-activation, short-range stiffness enhancement, and other history-dependent processes that accompany the stretch-to-shortening transition [18,19,20].

Finally, once θ crosses back over the latch angle, the main joint spring fires toward the target equilibrium angle with a velocity-dependent gain,

(11) $$\tau_{\mathrm{fire}}\left(\theta \right)=k\;f\left(\omega_{\mathrm{in}}\right)\;\left(\theta^{\mathrm{eq}}-\theta \right),\;f\left(\omega_{\mathrm{in}}\right)=1+\min\left(g_{\max},\beta \left|\omega_{\mathrm{in}}\right|\right),$$

where $\omega_{\mathrm{in}}$ is the angular velocity of the joint at the instant it first entered the stretched region and $g_{\max}$ caps the maximum stiffness enhancement. The parameter β converts the speed of the incoming pre-stretch into an effective firing gain; it is therefore the second athlete-specific mechanical coordinate of the reduced SSC response.

The sequence $\tau_{\mathrm{free}}\to \tau_{\mathrm{stretch}}\to \tau_{\mathrm{rebound}}\to \tau_{\mathrm{fire}}$ constitutes the complete active-joint model used for the shoulder and wrist. The latch geometry and bump stiffness define the mechanical pre-stretch window; the athlete-dependent SSC enhancement itself is represented by α and β (with the fixed saturation convention $g_{\max}$ used in the numerical examples). The model therefore reproduces the qualitative signature of a genuine SSC—passive loading during the stretch, active stiffening at the moment of reversal, and enhanced velocity-dependent release—while remaining low-dimensional enough to be merged with the delay-programmed kinetic chain. The parameter values used in the representative SSC simulations are listed in Table 3. The bump stiffness is taken as five times the baseline stiffness of each joint, and the reference angular velocities $\omega_{\mathrm{ref}}$ reflect the characteristic pre-stretch rates of the shoulder and wrist, so that $\beta =g_{\max}/\omega_{\mathrm{ref}}$ maps a typical eccentric entry velocity onto the maximum 10% firing gain.

#### 2.2.3. Energy Input by Stiffness Modulation

The active-joint model injects mechanical energy at two identifiable events, which can be quantified exactly. Let $\delta =\theta -\theta_{L}$ denote the compression of the bump spring. When the effective stiffness changes from $k_{\mathrm{bump}}$ to $\left(1+\alpha \right)k_{\mathrm{bump}}$ at constant δ, the instantaneously stored potential energy increases by

(12) $$\Delta E_{\mathrm{rebound}}=\frac{1}{2}\alpha k_{\mathrm{bump}}\delta^{2},$$

which we interpret as active input expressed through an increase in effective joint stiffness rather than through an explicit muscle force term. Similarly, when the main spring fires with gain $f(\omega_{\mathrm{in}})>1$, the elastic energy available to accelerate the joint toward $\theta^{\mathrm{eq}}$ increases by

(13) $$\Delta E_{\mathrm{fire}}=\frac{1}{2}k\left[f(\omega_{\mathrm{in}})-1\right]{\left(\theta^{\mathrm{eq}}-\theta \right)}^{2}.$$

The model therefore admits explicit energy bookkeeping: between the two active transitions the mechanical system evolves conservatively under Equation (6), while at each transition a well-defined quantity of active energy, $\Delta E_{\mathrm{rebound}}$ or $\Delta E_{\mathrm{fire}}$, is injected into the joint. This bookkeeping is what allows the effect of changing α and β to be isolated cleanly from the timing contribution of the kinetic chain.

#### 2.2.4. Why α and β Are Useful Mechanical Variables

Representing SSC action through the two active parameters α and β offers four practical advantages. First, it enters the rigid-body equations of motion directly through the joint torque, requiring no additional state beyond $\left(\theta ,\dot{\theta }\right)$ and a small number of history flags; any simulation built on Equation (6)—or on its spatial generalization—can adopt it as a drop-in replacement for a passive joint spring. Second, being low-dimensional, the parameters are in principle identifiable from athlete-specific joint-level motion data far more readily than the parameters of a multi-muscle Hill-type model, in the same spirit in which an effective leg or joint stiffness has long been used to characterize elastic behavior in running and hopping [31,32]. Third, the model naturally reproduces the empirical observation that a stronger or faster pre-stretch increases the power available during the subsequent shortening phase. Fourth, it predicts that different athletes should require different optimal preparatory postures: a player with large α and β stands to gain more from a pronounced pre-stretch, whereas a player with small α and β may be better served by a posture optimized primarily for passive elastic release and delay timing. This athlete-specific flexibility, rather than the size of the enhancement reported for any single illustrative parameter set, is the central motivation for representing SSC action through α and β instead of a single fixed universal enhancement factor: whatever the individually variable magnitude of an athlete’s active SSC contribution may be, it can be mapped onto these two joint-torque parameters and optimized within the same passive delay-programmed dynamics of Section 3.1.

## 3. Results

### 3.1. Delay-Programmed Firing Without SSC

#### 3.1.1. Elastic Energy and Latch Angles

The baseline firing model uses constant torsional spring constants

(14) $$k_{b}=300\;N\;m\;rad^{-1},\;k_{a}=100\;N\;m\;rad^{-1},\;k_{r}=10\;N\;m\;rad^{-1}.$$

These values are not fitted to dynamometric measurements from a specific athlete; following the strategy of our earlier pendulum studies [14,15], they are chosen to reproduce, at an order-of-magnitude level, the well-documented proximal-to-distal decrease in maximal joint-torque capacity: the trunk/hip complex sustains the largest restoring torque, the shoulder an intermediate torque, and the wrist the smallest. This ordering, $k_{b}>k_{a}>k_{r}$, is qualitatively consistent with segmental descriptions of proximal-to-distal torque and timing in throwing and striking motions [1,2,4] and with simple optimal-timing analyses of muscle activation in throwing-like tasks [33]. The qualitative conclusions drawn below—the ranking of single-joint speeds, the existence of an optimal release delay, and the superadditive character of multi-joint release—depend on this ordering and on the associated inertial hierarchy of the segments, not on the third significant figure of $k_{i}$. The stiffness constants should therefore be interpreted as reduced-model control parameters rather than measurements of subject-specific in vivo joint stiffness. No systematic stiffness-sensitivity analysis was performed in the present study. Accordingly, the reported numerical optima demonstrate the mechanical feasibility of delay-programmed energy transfer under the prescribed stiffness hierarchy, but do not establish invariance of the absolute speeds or optimal delays under arbitrary changes in stiffness magnitude or ranking. Establishing experimentally supported stiffness ranges and performing a systematic sensitivity analysis are important subjects for future work.

The target equilibrium posture is

(15) $$\theta_{b}^{\mathrm{eq}}=0^{\circ },\;\theta_{a}^{\mathrm{eq}}=30^{\circ },\;\theta_{r}^{\mathrm{eq}}=-30^{\circ },$$

so that the absolute racket angle at the target is approximately horizontal, $\phi_{r}^{\mathrm{eq}}=0^{\circ }$. This configuration is representative of the extended-arm, slightly laid-back-wrist posture reported at, or immediately before, ball contact in the tennis serve and forehand drive [3,8,9,10,34]. Its role in the model is not merely descriptive: $v_{\mathrm{tip}}$ is evaluated as each active joint angle $\theta_{i}\left(t\right)$ passes through $\theta_{i}^{\mathrm{eq}}$, and the firing-time optimization of Section 3.1.3 and Section 3.1.4 is a search for the set of release delays that brings every active joint through its own $\theta_{i}^{\mathrm{eq}}$ with maximum angular velocity at essentially the same instant—the same frame. This construction is the model-level analogue of the moment of maximal segment extension used to characterize efficient timing in our earlier pendulum-based descriptions of the kinetic chain [14,15].

The initial latched posture is

(16) $$\theta_{b}^{0}=-45^{\circ },\;\theta_{a}^{0}=-30^{\circ },\;\theta_{r}^{0}=-100^{\circ },$$

representing an exaggerated, maximally “cocked” backswing: the trunk counter-rotated away from the target, the upper arm drawn behind the trunk, and the racket laid back sharply relative to the forearm. Postures of this general character are the mechanical prerequisite for proximal-to-distal energy transfer documented in high-speed serves and ground strokes [2,3,4,6,7,35]. Within the model, $\theta_{i}^{0}$ is the configuration at which elastic energy is stored, in analogy with the eccentric pre-loading that precedes the concentric phase of a genuine SSC [18,19,20]; Section 2.2 makes this analogy explicit for the shoulder and wrist.

The stored elastic energy at joint *i* is

(17) $$E_{i}=\frac{1}{2}k_{i}{\left(\theta_{i}^{\mathrm{eq}}-\theta_{i}^{0}\right)}^{2},$$

giving

(18) $$E_{b}=92.53\;J,\;E_{a}=54.83\;J,\;E_{r}=7.46\;J,\;E_{\mathrm{total}}≃154.82\;J.$$

The essential modeling assumption is that each joint can be latched and released at an independently chosen time. Once released, joint *i* produces the restoring torque

(19) $$\tau_{i}=k_{i}\left(\theta_{i}^{\mathrm{eq}}-\theta_{i}\right);$$

equivalently, $\tau_{i}=-k_{i}\left(\theta_{i}-\theta_{i}^{\mathrm{eq}}\right)$, the standard sign convention for a linear restoring torque that drives $\theta_{i}$ toward $\theta_{i}^{\mathrm{eq}}$. prior to release it remains held at $\theta_{i}^{0}$, and if it is inactive in a reduced-degree-of-freedom simulation it is held at $\theta_{i}^{\mathrm{eq}}$. This construction deliberately separates two quantities that are inseparable in an unconstrained passive pendulum: (i) the amount of elastic energy available at each joint, fixed once $\theta_{i}^{0}$, $\theta_{i}^{\mathrm{eq}}$, and $k_{i}$ are specified, and (ii) the instant at which that energy is released, left as a free parameter—the firing time, or equivalently the release delay relative to the other joints. Because $v_{\mathrm{tip}}$ depends sensitively on the relative firing times once more than one joint is active, this separation is what allows the release delay to be optimized directly, rather than emerging implicitly from the passive coupled dynamics as in our earlier pendulum models [14,15].

#### 3.1.2. One Active Degree of Freedom

When only joint *j* is active, the remaining joints are held fixed at $\theta^{\mathrm{eq}}$ and the multi-body dynamics reduce, at the instant $\theta_{j}$ crosses $\theta_{j}^{\mathrm{eq}}$, to a single scalar energy balance,

(20) $$\frac{1}{2}k_{j}{\left(\theta_{j}^{\mathrm{eq}}-\theta_{j}^{0}\right)}^{2}=\frac{1}{2}M_{jj}^{*}\omega_{j}^{2},$$

where $M_{jj}^{*}$ is the effective scalar inertia of coordinate *j* evaluated at $\theta^{\mathrm{eq}}$. Hence

(21) $$\omega_{j}=\sqrt{2E_{j}/M_{jj}^{*}},$$

and substitution into Equations (4) and (5) gives a closed-form prediction for $v_{\mathrm{tip}}$ that can be compared directly with the numerically integrated trajectory (Table 2).

The shoulder-only case yields the highest single-joint speed. The shoulder spring stores substantial energy and acts through the long arm-plus-racket lever; the wrist has small effective inertia and can spin up rapidly, but its stored energy is limited by the small torque constant $k_{r}$; and the trunk, although it stores the largest energy of the three, rotates the entire chain rigidly about the base pivot rather than producing distal angular magnification, so much of its stored energy is converted into motion of the proximal mass rather than into racket-tip speed. No single joint, however, approaches the speeds reported for high-level strokes; the remaining sections show that the missing ingredient is timing, not stored energy.

#### 3.1.3. Two Active Degrees of Freedom

**Figure 3:**
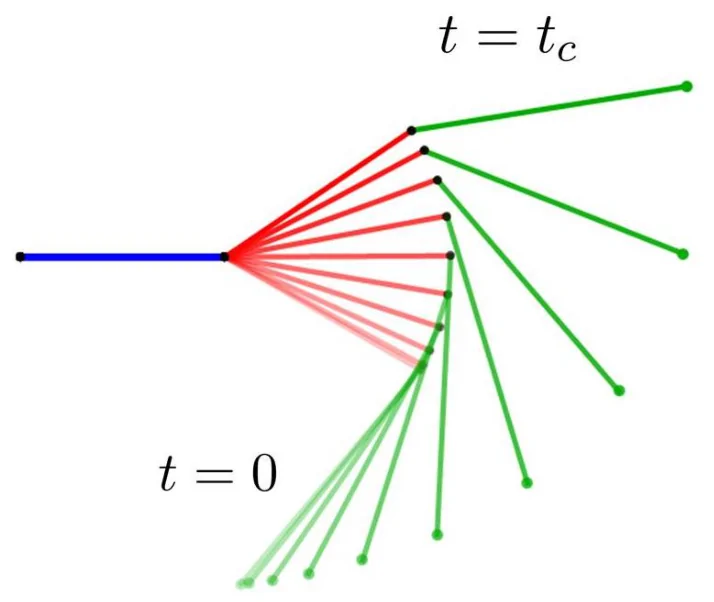
Two-degree-of-freedom (shoulder+wrist) release at the optimal delay Δt=0.040 s of Table 4, with the trunk held fixed at $\theta_{b}^{\mathrm{eq}}$ throughout. Ten superimposed configurations of the arm–racket linkage are shown at equal time intervals from t=0 (lightest, lower trace) to the impact time $t=t_{c}$ (darkest, upper trace). The shoulder joint releases its stored elastic energy at t=0; the wrist joint releases its own stored energy 0.04 s later. This lag allows the racket to begin accelerating only after the shoulder has already imparted substantial angular velocity to the arm, so that the racket tip attains its maximum speed close to $t=t_{c}$ rather than earlier in the motion.

With two active joints, the distal joint is released a time Δt after the proximal joint. Once two joints move simultaneously, the dynamics are no longer reducible to a single scalar energy balance, because the mass matrix M(q) depends on configuration and kinetic energy is continuously exchanged between coordinates through the off-diagonal terms $h_{12}$, $h_{13}$, $h_{23}$ (Appendix A). The qualitative mechanism, illustrated in Figure 3, is nevertheless straightforward: the proximal joint first accelerates the larger, slower segment, and the distal joint is then released into an already-moving mechanical environment. If the distal release occurs too early, the distal segment accelerates before the proximal segment has built up appreciable momentum, and the coupling benefit is lost; if it occurs too late, the distal segment reaches its own $\theta^{\mathrm{eq}}$ after the optimal hitting configuration has already been passed. An optimal, interior value of Δt therefore exists for every joint pair, and was located here by a one-dimensional numerical scan of $v_{\mathrm{tip}}\left(\Delta t\right)$ at the impact time (Table 4).

The shoulder–wrist pair produces the largest two-joint speed. The shoulder supplies a strong mid-chain acceleration over a comparatively large angular excursion, while the wrist, owing to its small effective inertia and long distal lever arm, converts a comparatively modest angular velocity into a rapid final “snap” of the racket, as visible in the final traces of Figure 3.

#### 3.1.4. Three Active Degrees of Freedom

With all three joints active, the trunk is fired at t=0 while the shoulder and wrist firing times, $t_{\mathrm{shoulder}}$ and $t_{\mathrm{wrist}}$, are treated as two independent free parameters. Because the three-joint dynamics couple through the full mass matrix M(q), no closed-form expression analogous to Section 3.1.2 is available; the optimal pair $\left(t_{\mathrm{shoulder}},t_{\mathrm{wrist}}\right)$ was instead located by a two-dimensional numerical search that maximizes $v_{\mathrm{tip}}$ at the impact time $t_{c}$. The resulting optimum is

(22) $$t_{\mathrm{shoulder}}=0.149\;s,\;t_{\mathrm{wrist}}=0.195\;s,$$

giving

(23) $$v_{\mathrm{tip},\max}=43.122\;m\;s^{-1},$$

a value that falls within the range reported for high-level tennis strokes and serves [3,5,34]. More significant than the absolute value is that the optimization has discovered, purely numerically, a firing schedule in which the trunk begins moving first, the shoulder is released only after the trunk has accumulated angular momentum, and the wrist/racket is released last, once the arm itself is moving rapidly—precisely the proximal-to-distal sequence associated with the kinetic chain. At the optimum, all three joints pass through their equilibrium angles with maximal angular velocity at essentially the same frame; when the delays are tuned in this way, effectively all of the recoverable stored energy is channeled into racket motion at impact.

Each optimal delay found here plays the same physical role as the sequential release timing analyzed in our earlier triple-pendulum study, where allowing the relative timing of segment release to be controlled explicitly was shown to increase the distal-segment speed beyond what a purely passive kinetic-chain release could achieve [15]. The delay-optimization procedure used here is the active-control counterpart of that mechanism: energy stored during the backswing is not allowed simply to propagate outward according to the natural passive dynamics of the coupled links, but is deliberately released joint by joint at the instant that best exploits the growing angular velocity of the more proximal segments. In this sense, the 1-DOF, 2-DOF, and 3-DOF results show how the kinetic chain is constructed mechanically by time-delayed joint motion.

**Figure 4:**
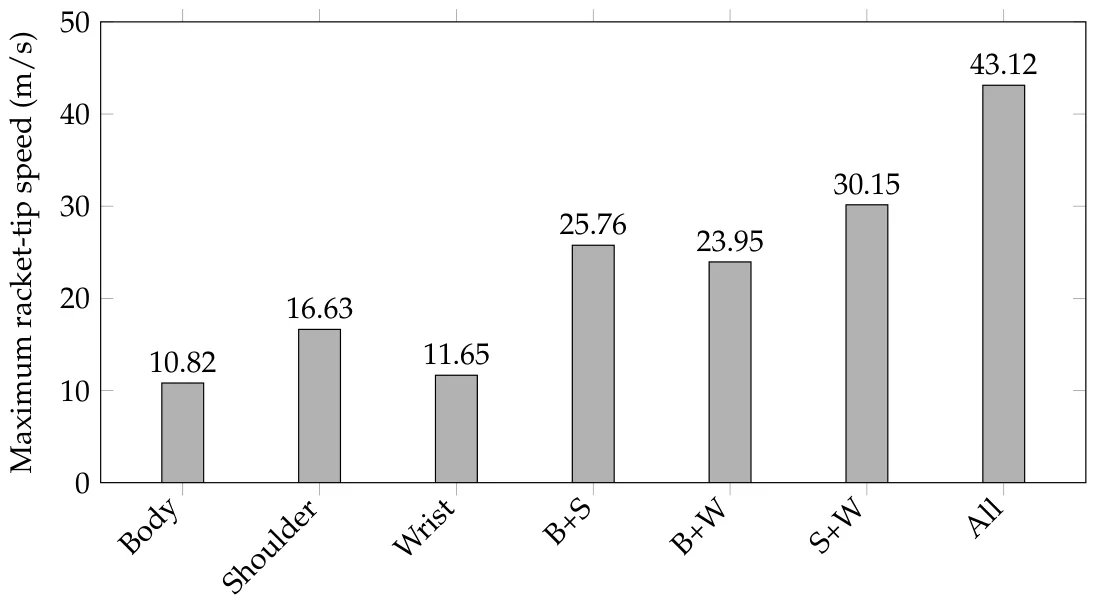
Maximum racket-tip speed for every non-SSC firing configuration studied in Section 3.1. Increasing the number of correctly delayed active degrees of freedom increases racket speed superadditively; the largest speed is reached only when trunk, shoulder, and wrist fire sequentially with the optimized delays of Section 3.1.4.

Figure 4 collects all of the non-SSC firing results. The progression from the best single-joint case (16.63 m s^−1^, shoulder) to the best two-joint case (30.15 m s^−1^, shoulder+wrist) to the three-joint case (43.12 m s^−1^) is markedly superadditive: the three-joint speed exceeds twice the best single-joint speed, even though only one further degree of freedom (the trunk) was added relative to the best two-joint case. This nonlinear scaling is the numerical signature of the kinetic chain: each additional proximal segment, when correctly timed, does not simply contribute its own share of racket speed but multiplies the effectiveness of the more distal segments by delivering them into motion with a higher base angular velocity before they, in turn, fire.

### 3.2. Adding the SSC Block to the Delay-Optimized Kinetic Chain

The combined simulations use the fully optimized three-joint firing schedule of Section 3.1.4 unchanged, with the shoulder and wrist joints now governed by the four-phase torque sequence of Section 2.2.2 and the parameters of Table 3. The schedule is deliberately not re-optimized jointly with α, β, and the pre-stretch velocities: the purpose of this comparison is to show that the SSC block can be added on top of an already delay-optimized passive schedule without disturbing it, not to claim that the reported schedule is itself jointly optimal once SSC is active. Searching jointly over the delays, α, β, and the pre-stretch velocities is a straightforward extension of the same numerical procedure and is left for future work. The four cases in Table 5 differ only in the imposed initial shoulder and wrist angular velocities at t=0; a negative velocity corresponds to the arm or racket still moving backward, i.e., an imposed pre-stretch or additional backswing loading, at the reference instant.

Three features of Table 5 deserve emphasis. First, relative to the non-SSC optimized three-joint result, $v_{\mathrm{tip},\max}=43.12\;m\;s^{-1}$, the representative SSC model with both joints pre-stretched reaches $v_{\mathrm{tip},\max}=46.74\;m\;s^{-1}$, a relative enhancement of

(24) $$\frac{46.74-43.12}{43.12}\times 100\%≃8.4\%.$$

This enhancement can be decomposed using the cases already listed in Table 5, without any further simulation. The “No imposed pre-stretch” case already activates the full four-phase SSC torque law, including the active rebound gain α, but imposes zero initial shoulder/wrist angular velocity; it reaches $v_{\mathrm{tip}}=42.95\;m\;s^{-1}$, within 0.5% of the passive, non-SSC optimum of 43.12 m s^−1^. Because the velocity-dependent firing gain of Equation (11) satisfies $f(\omega_{\mathrm{in}})\to 1$ as $\omega_{\mathrm{in}}\to 0$, this comparison shows that the active rebound gain α alone, without an imposed pre-stretch velocity, changes the peak speed by less than 0.5%. Essentially, the entire 8.4% enhancement is therefore produced once a nonzero pre-stretch velocity is imposed together with the velocity-triggered gain β (the “Shoulder”, “Wrist”, and “Both pre-stretched” rows). This is not a confound to be removed from the model but the mechanical signature the model is designed to reproduce: the physiological SSC is likewise known to depend on stretch *velocity* rather than on stretch magnitude alone [18,19]. The point of this result is accordingly not the precise size of the 8.4% enhancement, which is athlete-dependent and fixed here only for illustration, but the demonstration that a velocity-triggered active contribution of arbitrary, individually variable size can be folded into the already delay-optimized passive kinetic-chain model through α and β alone, without modifying the optimization architecture of Section 3.1. This number should be read only as the consequence of the illustrative parameter set in Table 3. It is not a universal SSC effect, nor is it the central claim of the paper. If α and β are changed, the speed increase changes accordingly. The important point is that such changes can be represented inside the same mechanical equations of motion that already produce the delay-optimized kinetic chain.

Second, the impact time shifts by no more than 3 ms across all four cases: for this parameter set, the SSC block adds speed within the delay-optimized firing schedule, so that the maximum racket speed is attained at essentially the same frame at which the kinetic-chain optimization alone would place it. Active stiffness modulation and delay programming are therefore not competing mechanisms requiring separate models; the same scan that optimizes the kinetic chain can also be used after specifying, or varying, the SSC parameters α and β. Third, the wrist pre-stretch contributes more than the shoulder pre-stretch for the parameter set used here. This asymmetry is expected on physical grounds: the wrist acts closest to the racket and has the smallest effective inertia among the three joints, so a modest active stiffness increase at the wrist is amplified, through the same lever effect responsible for the single-joint ranking of Table 2, into a comparatively large distal angular velocity.

**Figure 5:**
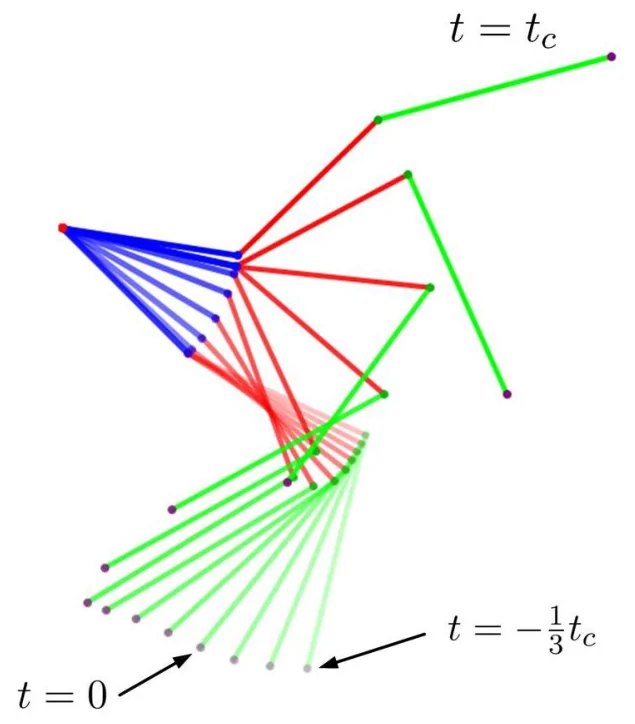
SSC-enhanced swing with both shoulder and wrist pre-stretched, $({\dot{\theta }}_{a}\left(0\right),{\dot{\theta }}_{r}\left(0\right))=\left(-2,-4\right)$ rad/s, and initial angles $\theta_{a}=12.0^{\circ }$, $\theta_{r}=-96.0^{\circ }$ at t=0. In contrast to Figure 3 and Figure 6, the configurations shown here begin *before* the reference instant, at $t=-t_{c}/3$, so as to display explicitly the backswing portion of the motion during which the arm and racket are still moving backward under the imposed pre-stretch condition. The sequence then proceeds through t=0 and on to the impact time $t=t_{c}$, again with ten configurations shown at equal time intervals. The initial convergence and subsequent reversal of the early, light-colored traces is the kinematic signature of the pre-stretch phase of the SSC ($\tau_{\mathrm{stretch}}$ in Section 2.2.2), which precedes the active rebound and main firing that ultimately accelerate the racket.

**Figure 6:**
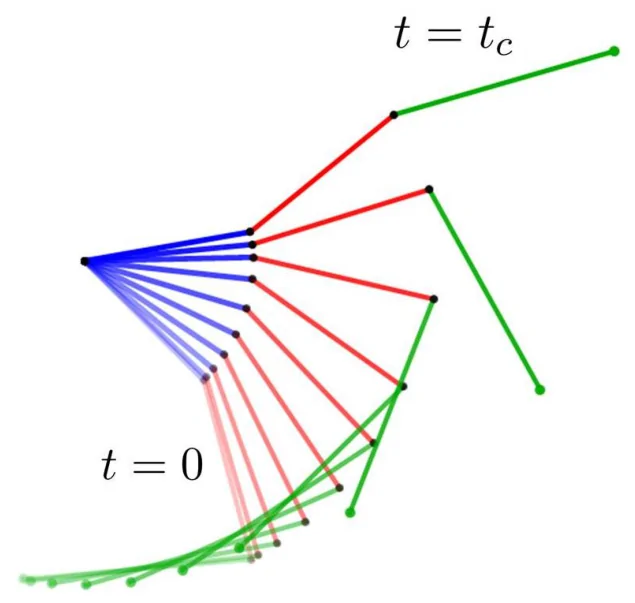
Fully optimized three-degree-of-freedom (body+shoulder+wrist) release. Using the same convention as Figure 3, ten superimposed configurations of the complete body–arm–racket chain are shown at equal time intervals from t=0 (lightest, lower trace) to the impact time $t=t_{c}$ (darkest, upper trace). The trunk rotates throughout the entire interval from t=0; the arm begins rotating relative to the trunk only after the shoulder release delay $t_{\mathrm{shoulder}}=0.149$ s; and the racket begins rotating relative to the arm only after the wrist release delay $t_{\mathrm{wrist}}=0.195$ s, i.e., 0.046 s after the shoulder. As a result, the racket orientation changes little during the early part of the motion and then sweeps through a large angle in the final fraction of the swing, producing the clearly visible whip-like acceleration that yields the maximum tip speed essentially at $t=t_{c}$.

The athlete-specific character of the model is also visible in the geometry of the motion itself. Comparing the SSC-enhanced swing of Figure 5 with the purely passive, optimized three-joint swing of Figure 6 reveals a noticeably different body–arm–racket trajectory, even though both simulations are optimized, in their respective senses, for maximum racket-tip speed. Different combinations of α, β, and pre-stretch velocity in general produce different optimal postures and visibly different swing shapes, each of which can nonetheless achieve close to maximal racket-tip speed for the corresponding parameter set. Thus the reduced model turns SSC from a qualitative physiological label into a pair of mechanical variables that can be scanned, fitted, or optimized inside the delay-programmed kinetic-chain framework.

## 4. Discussion

The central methodological claim of this paper is that racket-stroke optimization can be organized around a single mechanical idea: energy stored at successive joints should be released with appropriate time delays so that it is transferred efficiently through the kinetic chain to the racket. The 1-DOF, 2-DOF, and 3-DOF results demonstrate this principle directly. The SSC is then added not as a separate high-dimensional biological subsystem, but as a two-parameter stiffness-modulation block that can be inserted into the same joint-torque algorithm.

The delay-programmed model of Section 3.1 establishes the kinetic-chain result quantitatively: racket-tip speed increases from about 16.6 m s^−1^ for the best single active joint, to 30.2 m s^−1^ for the best pair, to 43.1 m s^−1^ once trunk, shoulder, and wrist are all released with optimized delays. This progression is not a numerical artifact of the optimization procedure; it follows directly from the inertial coupling encoded in the off-diagonal terms of M(q), the geometric leverage provided by the increasing distance of each successive segment from the base pivot, and the existence of a well-defined optimal release delay for each joint pair, as demonstrated analytically for the single-joint case and numerically for two and three joints. When the delays are tuned to their optima, every joint passes through its equilibrium angle with maximal angular velocity at essentially the same frame, and the stored energy is channeled almost entirely into racket motion at impact.

The SSC model of Section 2.2 then answers a separate but connected question: how can the physiological SSC be included without abandoning this delay-based kinetic-chain picture? The answer proposed here is to represent SSC action by the two effective parameters α and β. The representative simulations of Section 3 show that one illustrative parameter set raises the peak speed by roughly 8% at essentially the same impact frame. But the deeper result is not the percentage itself. The deeper result is that delay programming determines *when* stored energy is delivered through the kinetic chain, whereas α and β determine *how much additional effective stiffness and energy* are supplied by the SSC-like response. The two mechanisms are therefore composable within a single mechanical model. We emphasize that this composability does not depend on knowing, in advance, the numerical size of any individual athlete’s SSC contribution. Because α and β enter the model as ordinary joint-torque parameters, an athlete-specific SSC response of unknown and individually variable magnitude can be inserted into, and optimized within, the same delay-programmed kinetic-chain architecture already validated for the purely passive case of Section 3.1, rather than requiring a separate SSC-specific optimization scheme. The decomposition in Section 3 confirms that the model behaves consistently with this intent: the active rebound gain α alone is mechanically inert in the absence of pre-stretch velocity, so that the two parameters jointly, and not either one separately, stand in for the athlete’s active SSC capacity.

Several implications extend beyond the specific numbers reported here. Foremost, efficient racket acceleration is a problem of *timing* as much as of strength: a wrist fired too early cannot reach maximal speed because it fails to exploit the angular momentum already generated by the more proximal segments, whereas a correctly delayed release allows the racket to accelerate only once the trunk and arm have already been set in motion. Second, the SSC results indicate that the preparatory phase of the stroke—racket lag, shoulder loading, and the rapid reversal from backswing to forward swing—can be treated as a mechanical pre-stretch condition whose effect is controlled by α and β. Third, the optimal posture, timing, and even the outward shape of the swing should be expected to differ from one athlete to another [36]. Players with different α, β, and characteristic pre-stretch velocity are predicted to reach their own maximum racket speed through visibly different combinations of posture and timing, as illustrated by the marked difference between Figure 5 and Figure 6. This observation motivates a note of caution for coaching practice: because the swing shapes observed in professional footage reflect that athlete’s own strength, stiffness, and SSC parameters, the model raises the hypothesis—though it does not by itself constitute proof—that an amateur who reproduces only the outward form of a professional stroke, without comparable underlying SSC capacity, is not guaranteed the same racket-speed benefit. Fourth, because segment mass, length, joint stiffness, release timing, and SSC gains all appear as separate, explicit parameters, the model can be used to explore how changes in racket mass, arm length, or an athlete’s strength profile shift the optimal firing schedule—a feature of direct interest for equipment selection and racket–player matching in sports engineering.

These implications should be read in light of the model’s deliberate simplifications. The model is planar and excludes shoulder internal/external rotation, forearm pronation–supination, grip and string-bed compliance, aerodynamic drag, and the finite-duration ball–racket collision process. The baseline joint stiffnesses are likewise idealized reduced-model parameters rather than directly measured subject-specific quantities. Therefore, the present results should be read as demonstrating the proposed mechanism under a prescribed proximal-to-distal stiffness hierarchy, not as showing that the numerical optima are insensitive to all possible stiffness values or rankings. In an actual stroke, the finite contact time and the compliances of the ball, racket frame, string bed, and grip influence the contact force, energy transfer, and ball spin. These effects can in turn alter the release sequence that would be optimal for the coupled ball–racket system. The present conclusions are therefore explicitly limited to the generation of racket-head speed immediately before collision; they should not be interpreted as predictions of outgoing-ball speed, spin, or collision-specific optimal timing. The arm is a single rigid segment, and the SSC description is phenomenological, formulated at the level of net joint torque rather than resolved into individual muscle–tendon units as in full musculoskeletal frameworks [26,27,28]. The specific numerical values reported (parameter choices, optimal delays, enhancement percentages) should accordingly be regarded as illustrative of the underlying mechanisms rather than as normative targets. In addition, the SSC-enhanced results of Section 3 are reported for a single illustrative (α,β) pair rather than a systematic sensitivity map; mapping $v_{\mathrm{tip},\max}$ over a grid of (α,β) values, and jointly optimizing the release delays together with α, β, and the pre-stretch velocities (Section 2.1.4), are natural and computationally straightforward extensions of the present procedure that we leave for future work. We regard this simplicity, however, as the model’s principal virtue: by isolating a small number of physically distinct mechanisms—elastic storage, delayed release, inertial coupling, distal whip-like amplification, and active history-dependent stiffness modulation—the model renders the logic of efficient racket acceleration transparent while remaining explicit enough that each mechanism can be switched on or off, or its parameters varied, to test a specific hypothesis.

## 5. Conclusions

We have developed a minimal planar body–arm–racket model in which the kinetic chain is represented mechanically by stored joint energy and time-delayed joint release. Separating stored elastic energy from release timing converts the delay between successive joint releases into an explicit, optimizable parameter. For one active joint, the closed-form energy-conversion estimate matches the numerically integrated racket-tip speed, validating the numerical procedure. Optimizing the delays for two and three active joints produces a distinctly superadditive increase in racket-tip speed—from 16.6 m s^−1^ to 30.2 m s^−1^ to 43.1 m s^−1^—with all joints passing through their equilibrium angles at essentially the same frame. This is the quantitative expression of the kinetic-chain principle in the present model.

The second contribution is to show how the stretch–shortening cycle can be incorporated into this same kinetic-chain framework. Instead of treating SSC as a separate, highly detailed biological subsystem, we represent its net mechanical effect by a history-dependent joint stiffness law governed, in its active part, by two effective parameters: the rebound stiffness gain α and the velocity-dependent firing gain β. These parameters are intended to be athlete-specific mechanical descriptors, not universal biological constants. By varying or identifying α and β, SSC-like enhancement can be included in ordinary rigid-body dynamics and optimized together with stiffness, inertia, release delay, and posture.

For one illustrative pair of α and β, adding the SSC block to the delay-optimized three-joint kinetic chain raises the peak racket-tip speed to 46.7 m s^−1^ (≈8%) at nearly the same impact frame. This percentage is not a universal prediction; it changes with the chosen SSC parameters. Using the cases already reported in Table 5, we further verified that this enhancement requires a nonzero pre-stretch velocity: the active rebound gain α alone changes $v_{\mathrm{tip}}$ by less than 0.5%, confirming that it is the velocity-triggered gain β, and not a generic stiffness increase, that is responsible for the SSC-like benefit. The main conclusion is therefore methodological and mechanical: time-delayed joint release provides the base model for the kinetic chain, and the two-parameter SSC representation allows that base model to include athlete-specific pre-stretch effects without leaving the framework of standard mechanics. The resulting model provides an analytically transparent and computationally inexpensive tool for personalized swing optimization, training design, and racket–player matching. Because ball–racket collision is not modeled, these applications concern the pre-impact generation of racket-head speed only. Validation of outgoing-ball speed, spin, and collision-specific release timing requires a coupled compliant ball–string–racket–grip model.

## Figures and Tables

**Table 1:** Segment parameters used in the planar swing model.

Segment	Mass *m* (kg)	Length *l* (m)	Moment of Inertia *I*
Body/trunk	25.0	0.50	$m_{b}l_{b}^{2}/12$
Arm	3.0	0.55	$m_{a}l_{a}^{2}/12$
Racket	0.33	0.68	$m_{r}l_{r}^{2}/12$

**Table 2:** Single-active-joint (1-DOF) results. The closed-form energy-conversion estimate and the peak speed obtained from numerical integration of Equation (6) agree to the precision shown, confirming the internal consistency of the numerical model.

Active Joint	Initial Condition	Analytic $v_{\mathrm{tip}}$ (m/s)	Simulated $v_{\max}$ (m/s)
Body only	$\theta_{b}^{0}=-45^{\circ }$	10.815	10.815
Shoulder only	$\theta_{a}^{0}=-30^{\circ }$	16.631	16.631
Wrist only	$\theta_{r}^{0}=-100^{\circ }$	11.649	11.649

**Table 3:** Parameters used in the reduced SSC model.

Parameter	Shoulder	Wrist
Baseline stiffness *k* ($N\;m\;{\mathrm{rad}}^{-1}$)	100	10
Bump factor	5.0	5.0
$k_{\mathrm{bump}}$ ($N\;m\;{\mathrm{rad}}^{-1}$)	500	50
Active bump gain α	0.10	0.10
Maximum firing gain $g_{\max}$	0.10	0.10
Reference angular velocity $\omega_{\mathrm{ref}}$ ($\mathrm{rad}\;s^{-1}$)	20	80
$\beta =g_{\max}/\omega_{\mathrm{ref}}$ ($s\;{\mathrm{rad}}^{-1}$)	0.0050	0.00125

**Table 4:** Optimized 2-DOF results. For each joint pair, the release delay Δt of the distal joint relative to the proximal joint was scanned numerically, and the delay that maximizes $v_{\mathrm{tip}}$ at impact is reported together with the corresponding maximum speed.

Active Joints	Optimal Delay (s)	Maximum $v_{\mathrm{tip}}$ (m/s)
Body + shoulder	0.160	25.758
Body + wrist	0.107	23.948
Shoulder + wrist	0.040	30.156

**Table 5:** Representative SSC-enhanced results. All cases use the same firing schedule as the optimized non-SSC 3-DOF case of Section 3.1.4; only the imposed initial shoulder and wrist angular velocities differ.

Case	$\left({\dot{\theta }}_{a}\left(0\right),{\dot{\theta }}_{r}\left(0\right)\right)$ (rad/s)	Peak Speed (m/s)	Impact Time (s)
No imposed pre-stretch	(0,0)	42.95	0.218
Shoulder pre-stretch	(−2,0)	43.52	0.218
Wrist pre-stretch	(0,−4)	46.30	0.220
Both pre-stretched	(−2,−4)	46.74	0.221

## Data Availability

The simulation code and data supporting the findings of this study are available from the corresponding author on reasonable request.

## Appendix A. Mass Matrix and Coriolis Terms

Writing $c_{a}=\cos\theta_{a}$, $c_{r}=\cos\theta_{r}$, $c_{ar}=\cos\left(\theta_{a}+\theta_{r}\right)$, the symmetric mass matrix in Equation (6) is

(A1) $$M=\left[\begin{array}{ccc} h_{11} & h_{12} & h_{13}\\ h_{12} & h_{22} & h_{23}\\ h_{13} & h_{23} & h_{33} \end{array}\right],$$

with entries

(A2) $$\begin{array}{ll} h_{11} & =I_{b}+m_{b}{\left(\frac{l_{b}}{2}\right)}^{2}+I_{a}+m_{a}\left(l_{b}^{2}+{\left(\frac{l_{a}}{2}\right)}^{2}+l_{b}l_{a}c_{a}\right)\\ \; & \;+I_{r}+m_{r}\left(l_{b}^{2}+l_{a}^{2}+{\left(\frac{l_{r}}{2}\right)}^{2}+2l_{b}l_{a}c_{a}+l_{b}l_{r}c_{ar}+l_{a}l_{r}c_{r}\right), \end{array}$$

(A3) $$\begin{array}{ll} h_{12} & =I_{a}+m_{a}\left({\left(\frac{l_{a}}{2}\right)}^{2}+\frac{1}{2}l_{b}l_{a}c_{a}\right)+I_{r}\\ \; & \;+m_{r}\left(l_{a}^{2}+{\left(\frac{l_{r}}{2}\right)}^{2}+l_{b}l_{a}c_{a}+\frac{1}{2}l_{b}l_{r}c_{ar}+l_{a}l_{r}c_{r}\right), \end{array}$$

(A4) $$\begin{array}{cc} h_{13} & =I_{r}+m_{r}\left({\left(\frac{l_{r}}{2}\right)}^{2}+\frac{1}{2}l_{a}l_{r}c_{r}+\frac{1}{2}l_{b}l_{r}c_{ar}\right), \end{array}$$

(A5) $$\begin{array}{cc} h_{22} & =I_{a}+m_{a}{\left(\frac{l_{a}}{2}\right)}^{2}+I_{r}+m_{r}\left(l_{a}^{2}+{\left(\frac{l_{r}}{2}\right)}^{2}+l_{a}l_{r}c_{r}\right), \end{array}$$

(A6) $$\begin{array}{cc} h_{23} & =I_{r}+m_{r}\left({\left(\frac{l_{r}}{2}\right)}^{2}+\frac{1}{2}l_{a}l_{r}c_{r}\right), \end{array}$$

(A7) $$\begin{array}{cc} h_{33} & =I_{r}+m_{r}{\left(\frac{l_{r}}{2}\right)}^{2}. \end{array}$$

The Coriolis/centrifugal vector follows from the usual Christoffel-symbol construction,

(A8) $$c_{i}=\sum_{j=1}^{3}\sum_{k=1}^{3}\frac{1}{2}\left(\frac{\partial M_{ij}}{\partial q_{k}}+\frac{\partial M_{ik}}{\partial q_{j}}-\frac{\partial M_{jk}}{\partial q_{i}}\right){\dot{q}}_{j}{\dot{q}}_{k}.$$
